# Rupture of the Liver

**Published:** 1900-05-19

**Authors:** 


### RUPTURE OF THE LIVER.

Lest the reports from South Africa in regard to gunshot wounds of the abdomen should lead to a rebound against operations in other cases of abdominal injury, we would draw attention to a very remarkable case of extensive rupture of the liver related by Mr. Thomas Cawardine,[1] of Bristol, in which a successful result followed laparatomy and stuffing of the wound in the liver with iodoform gauze, "three and three-quarter yards of gauze, several folds thick," being packed into the laceration and into the abdominal cavity below it. The injury was the result of a crush by a heavy weight against a wall. The patient was pulseless when the operation was performed, and was apparently kept alive by intravenous injection of saline fluid. On the abdomen being opened there was no free gas or odour, and the spleen was intact, but the liver was found to be extensively lacerated. The right rectus was therefore cut across, and a large quantity of clot was turned out of the abdominal cavity. The whole hand could then be introduced into a laceration of the liver, which extended "right across the under surface from about the position of the gall-bladder—which was not to be seen—to the posterior part, dividing the liver almost in twain, and leaving the upper part of the capsule floating on the blood-clot. The remainder of the right lobe appeared to be badly smashed, and could not be defined owing to its crushed condition." Yet, notwithstanding this desperate injury, the man recovered. After the operation nearly a quart of saline fluid was poured into the abdominal cavity. The patient remained pulseless for 30 hours after the operation. Two months after the injury he went to the convalescent home, from which he returned looking fat and well, with the wound securely healed. Mr. Cawardine is much to be congratulated on his success.

[1] Lancet, May 12.

